# Protocol for treatment of Achilles tendon ruptures; a systematic review with network meta-analysis

**DOI:** 10.1186/s13643-018-0912-5

**Published:** 2018-12-23

**Authors:** Brad Meulenkamp, Dawn Stacey, Dean Fergusson, Brian Hutton, Risa Shorr MLIS, Ian D. Graham

**Affiliations:** 1 Faculty of Medicine, University of Ottawa, 1053 Carling Ave. Suite J129, Ottawa, ON K1Y 4E9 Canada; 2 Faculty of Health Sciences, Centre for Practice-Changing Research, The Ottawa Hospital Research Institute, University of Ottawa, Ottawa, Canada; 3 Department of Medicine, Centre for Practice-Changing Research Clinical Epidemiology Program, The Ottawa Hospital Research Institute, University of Ottawa, Ottawa, Canada; 4 Clinical Epidemiology Program, Centre for Practice-Changing Research, The Ottawa Hospital Research Institute, Ottawa, Canada; 5 The Ottawa Hospital, Ottawa, Canada; 6 School of Epidemiology and Public Health; Clinical Epidemiology Program, Centre for Practice-Changing, The Ottawa Hospital Research Institute Research, University of Ottawa, Ottawa, Canada

**Keywords:** Achilles tendon rupture, Systematic review, Network meta-analysis, Orthopedics, Surgery, Physiotherapy

## Abstract

**Background:**

Achilles tendon ruptures are a common injury and are increasing in incidence. Several management strategies exist for both non-operative and operative care, with each strategy offering unique risks and benefits. Traditional pairwise meta-analyses have been performed to compare management strategies; however, all treatment options have never been integrated in a single analysis. Network meta-analysis (NMA) is a generalization of pairwise meta-analysis, which allows for the comparison of multiple interventions based on all available direct and indirect evidence. The objectives of this review are to synthesize the evidence on the management options for acute Achilles tendon rupture and identify which treatment gives the best functional outcomes.

**Methods:**

A systematic review with NMA is planned. An electronic literature search will be performed in conjunction with an experienced information specialist in MEDLINE, EMBASE, CINAHL, PEDro, and the Cochrane Central Register of Controlled Trials. We will include randomized controlled trials with a minimum 6-month follow-up. Two independent reviewers will screen citations for eligibility, extract study data, and perform risk of bias assessments. The primary outcome will be disease-specific functional outcome scores (AOFAS, Leppilahti, modified Leppilahti) at 1 year. Secondary outcomes will include complications (re-rupture, sural nerve injury, wound complications, deep infection, secondary surgeries), strength, range of motion, return to work, return to sport, and quality-of-life measures (including the SF-36 questionnaire). Traditional pairwise meta-analyses will be performed for all direct comparisons where evidence is available, and NMAs will subsequently be performed where possible to compare all management strategies.

**Discussion:**

The data generated from this review will provide health-care providers with a clear evidence synthesis of all Achilles tendon rupture management strategies. Additionally, these data will be incorporated into the development of a patient decision aid to assist patients and clinicians in making a preference-based decision when faced with an Achilles tendon rupture.

**Systematic review registration:**

PROSPERO CRD42018093033.

**Electronic supplementary material:**

The online version of this article (10.1186/s13643-018-0912-5) contains supplementary material, which is available to authorized users.

## Background

The Achilles tendon is the most commonly ruptured tendon, with an increasing annual incidence of up to 40 per 100,000 person-years [1]. These injuries are traditionally most common in the male, “weekend warrior” population between ages 30–50 [2]. More recent studies, however, have demonstrated the incidence is rising in all age demographics up to the sixth decade of life as the population strives to remain active for longer [3, 4]. The optimal treatment of acute ruptures has long been debated, with both surgical and non-surgical options presenting unique risks and benefits. The most recent guidelines from the American Academy of Orthopedic Surgeons were only able to conclude a “limited” strength of recommendation for either operative strategies, and an “inconclusive” recommendation when choosing a non-operative strategy [5]. Accordingly, there is substantial practice variation amongst surgeons treating this injury.

Historically, non-operative management has been associated with a higher risk of tendon re-rupture. For this reason, many surgeons have advocated for operative treatment. Unfortunately, due to a tenuous soft-tissue envelope over the Achilles tendon, surgery may result in devastating wound complications and infections [2, 6]. As such, alternative management strategies have been sought to minimize the risks that come with both operative and non-operative care. In many centers, non-operative care has evolved to include early mobilization and functional rehabilitation [3, 4, 7]. This strategy has been shown to decrease the re-rupture rate to that similar to operative management [6]. Functional rehabilitation programs, however, do require significant patient engagement and access to physiotherapy for optimal results, which may present a barrier to some patient populations [8]. There is also concern that calf strength remains weaker with functional rehabilitation than with operative treatment, leading some to advise surgery for more active patients [9, 10]. Surgical care is evolving, with minimally invasive and percutaneous surgical techniques being developed to negate the risk of wound complications and infections found with open surgery. These techniques are more challenging than traditional open surgery, with a learning curve for surgeons, and are not yet widely used [11].

As treatment recommendations and strategies evolve, decisional conflict may arise when patients with Achilles tendon ruptures are faced with the need to choose a management option. There have been several reviews of management strategies, primarily focused on pairwise comparisons between individual operative and non-operative strategies [6, 12, 13]. However, to date, there has not been a comprehensive review comparing all available interventions together collectively in a unified analysis. Network meta-analysis (NMA) represents a generalization of traditional pairwise meta-analysis which allows for the comparison of multiple treatment alternatives based upon all available direct and indirect evidence [14–16]. To address knowledge gaps regarding the comparative effectiveness of surgical and non-surgical interventions for acute Achilles tendon ruptures, a systematic review incorporating NMAs will be performed. The question of interest for this review is framed as follows: In adult patients with acute Achilles tendon rupture, which operative or non-operative treatment strategy provides patients with the best functional outcomes and what treatment method results in the lowest rate harm endpoints including tendon re-rupture, wound-healing problems, re-operation, and others?

## Methods/design

A systematic review incorporating network meta-analyses will be conducted with methods guided by the Cochrane Handbook [17]. This protocol has been reported in accordance with the Preferred Reporting Items for Systematic review and Meta-Analysis Protocols (PRISMA-P) guidelines [18], and the review will be reported in adherence with the PRISMA extension statement for incorporating network meta-analysis [19]. A completed PRISMA-P checklist for the current review is provided in Additional file 1. This protocol has been registered with PROSPERO CRD42018093033. Any deviations from the methods described will be reported in the final review.

### Eligibility criteria

Detailed eligibility criteria have been developed following the Population, Intervention, Comparator, Outcomes, and Study Design (PICOS) format [20]. These are described below in detail and summarized in Table 1.

**Table 1:** Inclusion and exclusion criteria

	Inclusion	Exclusion
Population	• Adult patients, > 16 years • First acute Achilles tendon rupture (< 4 weeks)	• Pediatric patients • Chronic rupture (> 4 weeks) • Re-rupture • Achilles tendonopathy • Musculotendinous junction tears
Intervention	• Open surgical management	• All other intervention types
Comparator	• Non-operative care, standard rehabilitation • Non-operative care, functional rehabilitation • Percutaneous surgery	• All other intervention types
Outcome	Primary • Disease-specific functional outcome measures Secondary • Overall complications • Re-rupture • Sural nerve injury • Wound complications • Deep infection • Secondary surgery • Return to work or sport • Strength • Range of motion • Quality-of-life instruments	• Less than 6-month follow-up • Outcome of interest not reported
Study designs	• Randomized controlled trials	• Observational studies • Studies reported only in the following forms: letters; commentaries, review articles • Abstracts with incomplete data

#### Population

This study will include adult patients over the age of 16 years treated for an acute, first-time Achilles tendon rupture. Specific population exclusion criteria will include pediatric patients, chronic tendon ruptures, tendon re-ruptures, patients with a documented history of Achilles tendonopathy, and musculotendinous junction ruptures. The exclusions have been chosen as these features may alter natural history of tendon repair and patient rehabilitation, resulting in differential effectiveness of the treatment strategies of interest.

#### Intervention

The reference intervention is an open surgical treatment using a longitudinal surgical approach. We hypothesize this to be the superior treatment method with respect to functional outcomes.

#### Comparators

Alternative treatment options for acute Achilles tendon rupture of interest will include: (1) non-operative care with cast and/or boot immobilization, (2) non-operative care with functional rehabilitation, and (3) percutaneous or minimally invasive surgery (MIS). These treatments will make up the network comparators. Functional rehabilitation will be defined as initiation of ankle range of motion prior to 6 weeks post-rupture. Percutaneous or MIS treatment will include all surgical modalities that do not completely open and reflect paratenon, including limited transverse incisions, suture-shuttling techniques, and device-assisted techniques.

#### Outcomes

Our primary outcome is disease-specific functional outcome at 1 year, measured using any of several Achilles-specific or general functional outcome measures including the American Orthopedic Foot and Ankle Score (AOFAS) [21], Leppilahti/Modified Leppilahti score [22], or others. Secondary outcomes of interest will include; overall complication rate, re-rupture rate, sural nerve injury, wound complication rate, deep infection rate, secondary surgery rate, strength, range of motion, return to work, return to sport, Short-Form 36, or other general quality-of-life instruments. Time to endpoints will be evaluated as such, with all others evaluated at 1-year.

#### Study designs

To minimize bias and methodological heterogeneity, only randomized controlled trials with a minimum of 6-month follow-up will be included. This duration of follow-up has been chosen as several relevant outcome measures may not be established early in patient recovery, such as tendon re-ruptures, strength, and range of motion [23]. We will exclude all other study designs, as well as studies reported in the context of letters and abstracts.

### Search methods and information sources

We will perform a comprehensive electronic search of the medical and rehabilitation literature using medical subject headings (MeSH) and text related to management of acute Achilles tendon rupture. Electronic searches will be performed, from inception to present day, using MEDLINE, EMBASE, CINAHL, PEDro, and Cochrane Central Register of Controlled Trials. A content expert (BM) developed the search strategy in consultation with a senior information specialist (RS) and had it peer reviewed by a second medical librarian in accordance with the Peer Review of Electronic Search Strategies (PRESS) framework [24].

The specific search strategies will be modified as needed for the included electronic databases, with a sample MEDLINE search strategy outlined in Additional file 2. A second search will be performed using a filter for systematic reviews, and reference lists of selected review articles will be cross-referenced to identify any additional studies. We will also search ClinicalTrials.gov to include relevant trials in progress. Relevant gray literature will be searched, including meeting abstracts from the annual Orthopedic Trauma Association (OTA), American Academy of Orthopedic Surgery (AAOS), and American Orthopedic Foot and Ankle Society (AOFAS) from 2014 to 2017 to identify emerging studies nearing completion. We will attempt to contact authors of any pertinent unpublished studies to ensure complete data extraction; however, abstracts will be excluded if data remains incomplete. Non-English publications will be translated as needed.

### Study records

Search strategy results will be uploaded to the Covidence online systematic review platform (Veritas Health Information Ltd., Victoria, Australia). Two independent reviewers will screen all titles and abstracts to identify potentially eligible studies. The same two reviewers will then conduct full-text screening to identify studies meeting the inclusion/exclusion criteria. Reasons for excluding full texts will be documented both in Covidence and an Excel spreadsheet (Microsoft Corporation, Redmond Washington). Study authors will be contacted if eligibility criteria remain unclear following article review. Disagreements will be resolved via consensus where possible and by a third reviewer, if necessary. Final study inclusion will be presented in a PRISMA flow diagram [25]. In the instance of duplicate data due to study updates, only the most recently published data will be included unless additional relevant data is presented.

### Data extraction

A standardized data extraction form will be developed a priori. The first five included studies will be used to pilot the data extraction form and revisions will be made as needed based on reviewer feedback. Two reviewers will independently extract data from all included studies and compare at review completion. Discrepancies will be resolved by consensus or input from a third team member. The following data will be extracted: study author, year of publication, study size, the inclusion criteria, and outcomes as outlined in the PICOS.

We will also capture data points to evaluate heterogeneity and effect modifiers across studies. These will include patient age, sex, study location, length of follow-up, risk factors for complications (smoking status, fluoroquinilone or steroid use, diabetes, prior tendonopathy, or other), surgical repair method (type of suturing method and suture type), and surgeon experience with the performed surgical procedures, if reported. Specific details of rehabilitation protocols will be noted, including time to immobilization, type of immobilization method, time to partial and full weight-bearing, and time to initiate range of motion. Sources of funding will be collected as part of risk of bias assessment. Study authors will be contacted in the cases of incomplete data. All data will be compiled in a Microsoft Excel spreadsheet for analysis.

### Risk of bias assessment

Included studies will undergo a risk of bias and reporting quality assessment by two reviewers. The first five assessments will be piloted for agreement and disagreements resolved by consensus. A third reviewer will be consulted as needed. Study authors will be contacted as necessary when there remains uncertainty in methodology or results reporting. Randomized controlled trials will be assessed using the Cochrane Handbook’s Risk of Bias (ROB) assessment tool [26]. Studies will be reviewed and scored as “high risk,” “low risk,” or “unclear” in each of the following domains: random sequence generation, allocation concealment, blinding of participants and personnel, blinding of outcome assessment, incomplete outcome data, selective reporting, and other bias. Inter-rater reliability of the ROB tool has been demonstrated to range from fair to substantial depending on the assessment domain [27]. The results of the risk of bias assessment will be summarized narratively with full assessments included in the appendix. Risk of bias between studies (publication bias, small sample size bias) will be assessed and presented as funnel plots [28].

### Data synthesis

A descriptive summary of pertinent study methodological and clinical characteristics will be initially reported. This will include summaries of key study and patient traits, included interventions, reported outcomes, and risk of bias assessments.

#### Pairwise meta-analysis

Meta-analysis using a random-effects model will be performed where studies are judged to be of adequate clinical, methodological, and statistical heterogeneity (*I*^2^ < 50%) [29]. For study-level and pooled results, dichotomous data will be expressed as odds ratios (OR) with 95% confidence intervals and continuous outcomes will be presented as mean differences (MD) with 95% confidence intervals. For functional outcomes where different scales are used, standardized mean differences (SMD) will be used. Analysis will be performed using the Cochrane Collaboration’s Review Manager Software (Version 5.3, The Cochrane Collaboration, Nordic Cochrane Centre, Copenhagen, Denmark).

#### Network meta-analysis

To compare all interventions, NMAs for each outcome are planned. The appropriateness of these analyses is based on the assumption of transitivity and exchangeability of studies. Theoretically, all patients in any given included study could have been randomizable to any of the management strategies of interest [30]. This assumes that there would be no absolute contraindication to any of the strategies for any given included patient. We will evaluate the transitivity assumption by comparing study inclusion and exclusion criteria, as well as patient demographic data including age, sex, time to treatment, and co-morbidities.

A Bayesian approach will be used in all analyses, with modeling guidance as described elsewhere from the National Institute for Health and Care Excellence guidelines [31–33]. Analyses will be performed using WinBUGS statistical software (Version 1.4.3, MRC Biostatistics Unit, Cambridge, UK) in a Markov Chain Monte Carlo framework with burn-in and sampling iterations of 20,000 or more. For each outcome of interest, both fixed- and random-effects models will be run. Adequacy of model fit of each analysis will be assessed by comparison of the total posterior residual deviance with the number of unconstrained data points (i.e., the number of intervention arms across studies in the analysis), as these quantities should be approximately equal. Choice between models will be based on evaluation of Deviance Information Criteria (DIC), with smaller values being preferred and a difference of 5 or more points being considered to represent an important difference in fit. Model convergence will be evaluated using the Gelman-Rubin diagnostic. Consistency will be evaluated by comparison of effect measures from pairwise meta-analyses with the corresponding NMA estimates, as well as by fitting inconsistency models to the data. For the latter, comparison of DIC with DIC from the corresponding consistency analysis and plotting of the deviance residuals from each model in a scatterplot will be performed to identify differences in magnitude that may be suggestive of inconsistency of direct and indirect evidence. If potential inconsistency is identified, we will explore the characteristics of the studies in the analysis and perform additional analyses to identify a remedy to resolve its presence.

Pairwise comparisons will be reported using the appropriate summary estimates with 95% credible intervals. Network geometry will be presented both with a network graph, descriptively summarizing interventions. Results will be presented using forest plots and/or league tables, as well as summarized in layperson’s language in the manuscript text. We will also present values of the Surface Under the Cumulative Ranking (SUCRA) curve for each treatment as well as treatment rankings [34].

### Sensitivity analysis and meta-regression

To examine the impact of bias on study results, sensitivity analyses will be performed excluding studies deemed at high risk of bias (studies with three or more categories ranked “high” on the risk of bias assessment). We will also use subgroup analysis and/or meta-regression to assess the effects of sources of heterogeneity if sufficient data exists. This will include subgroup analysis based on variation in rehabilitation protocols, and meta-regression to investigate the effect of patient co-morbidities and risk factors for Achilles rupture in studies (smoking, fluoroquinilone or steroid use, diabetes, and history of Achilles tendonopathy). As outcome data specific to these groups is unlikely to be reported, sensitivity analysis may be performed as an alternative excluding studies including a high proportion of patients with these characteristics. Results from all sensitivity analyses carried out will be discussed, with results provided in the supplement to the completed review for completeness.

## Discussion

Achilles tendon ruptures are increasing in incidence, and literature continues to accumulate for competing management interventions [8]. With the dissemination of information through online access, patients are more able than ever to access resources related to illness and injury. Each intervention comes with a unique set of benefits and harms. Whether the harms outweigh the benefits is a preference-sensitive decision, based not only on the expected outcomes but also on patient goals, values, and expectations. To date, only pairwise comparisons for management strategies for Achilles tendon rupture have been performed. As there are several strategies currently in practice, network meta-analysis methodology is an appropriate and powerful approach to synthesize available data and facilitate knowledge transfer to both clinicians and patients.

The primary goal of this network meta-analysis is to synthesize the full body of high-level evidence regarding Achilles tendon rupture management strategies. These data will be used in the development of a novel patient decision aid (PtDA). These tools translate evidence-based information on treatment options, risks, and harms to patients in language patients can understand. This allows patients to better merge personal values and priorities into treatment decisions, which have been demonstrated to improve patient engagement, satisfaction, and potentially clinical outcomes, [35].

In addition to adhering to a robust methodology as proposed in the NICE guidelines, the primary strength of this review will be in the completeness of the data acquisition and analyses. To our knowledge, this will be the first network meta-analysis to compare intervention strategies for Achilles tendon ruptures. With evolving management strategies, clinicians and patients face significant challenges when evaluating treatment options. Synthesizing the results of all treatment modalities and presenting results for all outcomes of clinical importance will greatly facilitate decision making for both parties. This study will contribute considerably to the advancement of evidence-based musculoskeletal care of patients with Achilles tendon rupture.

## Additional files

Additional file 1: PRISMA-P 2015 Checklist. (DOCX 38 kb)

Additional file 2: Search strategy. (DOCX 69 kb)

## Abbreviations

AAOS: American Academy of Orthopedic Surgery
AOFAS: American Orthopedic Foot and Ankle Score
DIC: Deviance Information Criteria
MD: Mean Differences
MeSH: Medical subject headings
MIS: Minimally invasive surgery
NMA: Network meta-analysis
OR: Odd’s ratio
OTA: Orthopedic Trauma Association
PICOS: Population, Intervention, Comparator, Outcome, and Study design
PRESS: Peer Review of Electronic Search Strategies
PRISMA: Preferred Reporting Items for Systematic review and Meta-Analysis
PRISMA-P: Preferred Reporting Items for Systematic review and Meta-Analysis Protocols
PtDA: Patient decision aid
ROB: Risk of bias
SMD: Standardized mean differences
SUCRA: Surface Under the Cumulative Ranking
