# Supplementary material for: Protocol for treatment of Achilles tendon ruptures; a systematic review with network meta-analysis
Source: Syst Rev. 2018 Dec 23;7:247. doi: 10.1186/s13643-018-0912-5 (PMC6304227; doi:10.1186/s13643-018-0912-5)
Supplement: Supplementary file 2 — Search strategy. (DOCX 69 kb) [file 13643_2018_912_MOESM2_ESM.docx]

Database: Ovid MEDLINE(R) ALL <1946 to July 25, 2018>

Search Strategy:

1 achilles tendon/ (16990)

2 (achill* or tendoachill* or calcaneal).tw. (37844)

3 1 or 2 (41306)

4 rupture/ or ruptur*.tw. (290970)

5 3 and 4 (6677)

6 ((achill* or tendoachill* or calcaneal) and (ruptur* or tear* or damag* or injur*)).kf. (389)

7 ((achill* or tendoachill* or calcaneal) adj3 repair*).tw. (1834)

8 ((achill* or tendoachill* or calcaneal) adj5 (tear* or damage* or injur*)).tw. (2010)

9 or/5-8 (8399)

10 randomized controlled trial.pt. (917562)

11 controlled clinical trial.pt. (182870)

12 random*.tw. or placebo.ab. (3209251)

13 clinical trials as topic.sh. (217426)

14 trial*.ti. (829576)

15 or/10-14 (3894307)

16 9 and 15 (886)

17 exp animals/ not humans/ (16581887)

18 16 not 17 (600)

19 18 use medall (267)

20 achilles tendon rupture/ (2444)

21 achilles tendon/ (16990)

22 tendon rupture/ (4380)

23 21 and 22 (299)

24 ((achill* or tendoachill* or calcaneal) and ruptur*).tw. (6062)

25 ((achill* or tendoachill* or calcaneal) adj3 repair*).tw. (1834)

26 ((achill* or tendoachill* or calcaneal) adj5 (tear* or damage* or injur*)).tw. (2010)

27 20 or 23 or 24 or 25 or 26 (8547)

28 double-blind*.mp. or placebo*.tw. or blind*.tw. (1300956)

29 random*.tw. (2991078)

30 randomized controlled trial/ (976028)

31 28 or 29 or 30 (3730276)

32 27 and 31 (913)

33 (exp animal/ or nonhuman/) not exp human/ (11143609)

34 32 not 33 (719)

35 34 use emczd (307)

36 achilles tendon/ (16990)

37 (achill* or tendoachill* or calcaneal).tw. (37844)

38 36 or 37 (41306)

39 rupture/ or ruptur*.tw. (290970)

40 38 and 39 (6677)

41 ((achill* or tendoachill* or calcaneal) adj3 repair*).tw. (1834)

42 ((achill* or tendoachill* or calcaneal) adj5 (tear* or damage* or injur*)).tw. (2010)

43 40 or 41 or 42 (8346)

44 43 use cctr (280)

45 19 or 35 or 44 (854)

46 remove duplicates from 45 (512)
